# Differential responses to genotoxic agents between induced pluripotent stem cells and tumor cell lines

**DOI:** 10.1186/1756-8722-6-71

**Published:** 2013-09-20

**Authors:** Yinghua Lu, Dazhong Xu, Jing Zhou, Yupo Ma, Yongping Jiang, Wenxian Zeng, Wei Dai

**Affiliations:** 1 College of Animal Science and Technology, Northwest A&F University, Yangling, Shaanxi, China; 2 Department of Environmental Medicine & Pharmacology, New York University Langone Medical Center, 57 Old Forge Road, Tuxedo, NY, USA; 3 Department of Pathology, The State University of New York, Stony Brook, USA; 4 Biopharmaceutical Research Center, Chinese Academy of Medical Sciences & Peking Union Medical College, Suzhou, China

**Keywords:** iPS cells, Genotoxic stress, DNA damage, Chromium, Phosphorylation, P53

## Abstract

Given potential values of induced pluripotent stem (iPS) cells in basic biomedical research and regenerative medicine, it is important to understand how these cells regulate their genome stability in response to environmental toxins and carcinogens. The present study characterized the effect of Cr(VI), a well-known genotoxic agent and environmental carcinogen, on major molecular components of DNA damage response pathways in human iPS cells. We compared the effect of Cr(VI) on human iPS cells with two established cell lines, Tera-1 (teratoma origin) and BEAS-2B (lung epithelial origin). We also studied the effect of hydrogen peroxide and doxorubicin on modulating DNA damage responses in these cell types. We demonstrated that ATM and p53 phosphorylation is differentially regulated in human iPS cells compared with Tera-1 and BEAS-2B cells after exposure to various genotoxic agents. Moreover, we observed that inhibition of CK2, but not p38, promotes phosphorylation of p53^S392^ in iPS cells. Combined, our data reveal some unique features of DNA damage responses in human iPS cells.

## Introduction

Induced pluripotent stem (iPS) cells are derived from somatic cells through genetic re-programming. Simultaneous ectopic expression of key transcription factors such as OCT4, SOX2, KLF4 and c-MYC can reprogram human somatic cells to pluripotent stem cells capable of differentiation into a variety of cell types [1]. Human iPS cells thus represent a valuable resource for the development of *in vitro* models for human diseases and have great potentials in regenerative medicine [2]. Recent studies have shown that human iPS cells also offer a valuable alternative to human embryonic stem cells for drug development [3], as well as for in vitro expansion and differentiation into cells of the hematopoietic lineage [4,5]. It is well known that cells undergoing *in vitro* expansion are constantly exposed to a variety of environmental insults including genotoxic agents and oxidative stress. Given the great potential of iPS cells, it is imperative to understand the characteristics of these cells, especially regarding their genomic stability after exposure to environmental genotoxic agents.

Chromium (VI) compounds are well established environmental carcinogens that produce genotoxic effects leading to human cancers [6-9]. Chromium (VI) generates reactive oxygen species (ROS) that induce DNA damage, which is thought to trigger DNA damage responses in somatic cells [6-8]. Although some studies have been carried out with an emphasis on toxic and carcinogenic effects of Cr(VI) compounds on somatic cells [7,8], its effect on human iPS cells remains largely unknown. In fact, very limited studies have been conducted on DNA damage responses caused by genotoxic agents in either embryonic stem cells or iPS cells. Cr(VI) has been shown to inhibit differentiation of murine embryonic stem cells [10]. A strong DNA damage response induced by γ-irradiation has been demonstrated in human iPS cells [11]. Given the unique chromatin structure of iPS cells, it is likely these cells may respond to DNA damage differently after challenge with genotoxic agents including Cr(VI) compared with those cells of the somatic origin.

The DNA damage response entails a series of signaling events including auto-phosphorylation of ATM and phosphorylation of histone H2AX and p53 [12,13]. Extensive research in the past has identified amino acid residues in these proteins that are characteristic of DNA damage responses [13-17]. They include ATM^S1981^, p53^S15^, p53^S20^, p53^S392^, and H2AX^S139^[13-17]. In the current study, we evaluated the effect of Cr(VI) on expression and/or activation of several key molecular components mediating DNA damage responses in human iPS cells and compared it with those of transformed cells from the somatic origin (Tera-1 and BEAS-2B). As additional controls, we also exposed these cells to H_2_O_2_ and doxorubicin (Dox), two well studied genotoxic agents. We found that human iPS cells responded differently to Cr(VI) compared with Tera-1 and BEAS-2B cells in terms of activation of DNA damage response pathway. In addition, we observed that iPS cells, Tera-1 and BEAS-2B exhibited differential responses after H_2_O_2_ or Dox treatment. Our findings indicate that iPS cells have some unique features to Cr(VI) and other genotoxic agents that can be explored for potential drug developments.

## Experimental procedures

### Cell lines and cell culture

Human induced pluripotent stem cells were derived from human amniotic fluid-derived cells (hAFDCs) via retrovirus-mediated expression of four transcription factors (OCT4/SOX2/KLF4/C-MYC). Human iPS cells were cultured in 6-cm tissue culture dishes coated with matrix (Invitrogen, USA) in a feeder-free culture conditions using Essential 8™ medium. Human iPSCs grown on feeder-dependent culture conditions (Mitomycin C treated murine embryonic fibroblasts) were maintained in DMEM-F12 (Invitrogen, USA) medium which was supplemented with 20% KSR, 10 ng/mL bFGF, 2 mM GlutaMAX™-I, 0.1 mM MEM Non-Essential Amino Acids Solution, 1 × β-mercaptoethanol. Cells were passed every 5–6 days after trypsinization. Mitomycin C treated murine embryonic fibroblasts (MEFs) were prepared as feeder cells. Tera-1 cells obtained from American Type Culture Collection (ATCC) were cultured in McCoy’s 5A medium supplemented with 10% fetal bovine serum (FBS). BEAS-2B cells obtained from ATCC were cultured in DMEM supplemented with 10% FBS.

### Antibodies

Antibodies to p53, NANOG and SOX2 (for flow cytometry) were purchased from Santa-Cruz Biotechnology. Antibodies to OCT4, SOX2 (for Western blot), ATM, phospho-ATM^S1981^ (p-ATM^S1981^), p-p53^S15^, p-p53^S20^, γ-H2AX, p-p53^S392^, p-p38, p38, PARP-1, α-tubulin, β-actin were purchased from Cell Signaling Technology.

### Fluorescence microscopy

Human iPS cells were fixed in 4% paraformaldehyde (PFA) and washed with PBS containing 5% BSA and 0.4% Triton X-100. These cells were subsequently incubated with antibodies against OCT4 at 37°C for 1 h followed by washing three times with PBS with Tween. Cells were then incubated with a secondary antibody conjugated with Alexa Fluor 555 at 37°C for 45 min followed by wash three times with PBS with Tween. After brief staining with Hoechst, cells were examined under a fluorescence microscope.

### Flow cytometry analysis

Human iPS cells were disaggregated by 0.5 mM EDTA, washed with PBS, and then treated with 4% PFA and 0.4% Triton X-100 for 30 min. After wash twice with cold PBS containing 0.5% fetal bovine serum, cells were incubated with an anti-human NANOG or SOX2 antibody for 30 min at 4°C. Subsequently, these cells were stained with a second antibody IgG conjugated with Alexa Fluor555 for 30 min. Isotype control IgG was used as control. The fluorescence-labeled cells were analyzed with flow cytometry.

### Differentiation of iPS cells

Human iPS cells were maintained in Essential 8^TM^ medium (Life Technologies). OP-9 cells were cultured in the MEMα medium containing 20% FCS. The iPS cells were induced to differentiate into embryonic stem cell sacs (ES-sacs) as described [18]. Briefly, the small clumps of human iPS cells were transferred onto OP-9 cells and cultured in a differentiation medium (IMDM supplemented with 10 μl/ml ITS 100× stock solution, 2 mM L-glutamine, 0.45 μM MTG, 50 μg/ml ascorbic acid, 15% FBS and 20 ng/ml recombinant human VEGF), which was refreshed every 3 days. On days 14 to 15 of culture, ES-sacs emerged. Representative images of ES-sacs were captured under microscope. Human iPS cells were induced to differentiate toward the neural lineage was carried out using STEMdiff™ Neural Induction Medium according to the manufacturer’s protocol (Stem Cell Technologies).

### Western blot

Human iPS cells treated with appropriate chemicals for various times were washed twice with ice-cold PBS and lysed in 310 μL RIPA buffer supplemented with protease and phosphatase inhibitors. Cell lysates were centrifuged by at 12,000 g for 15 min at 4°C. Equal amounts of cell lysates (20 μg) were mixed with 6× SDS loading buffer, heated at 100°C for 5 min, and analyzed by SDS-PAGE followed by Western blotting.

## Results

### Characterization of human iPS cells

**Figure 1:**
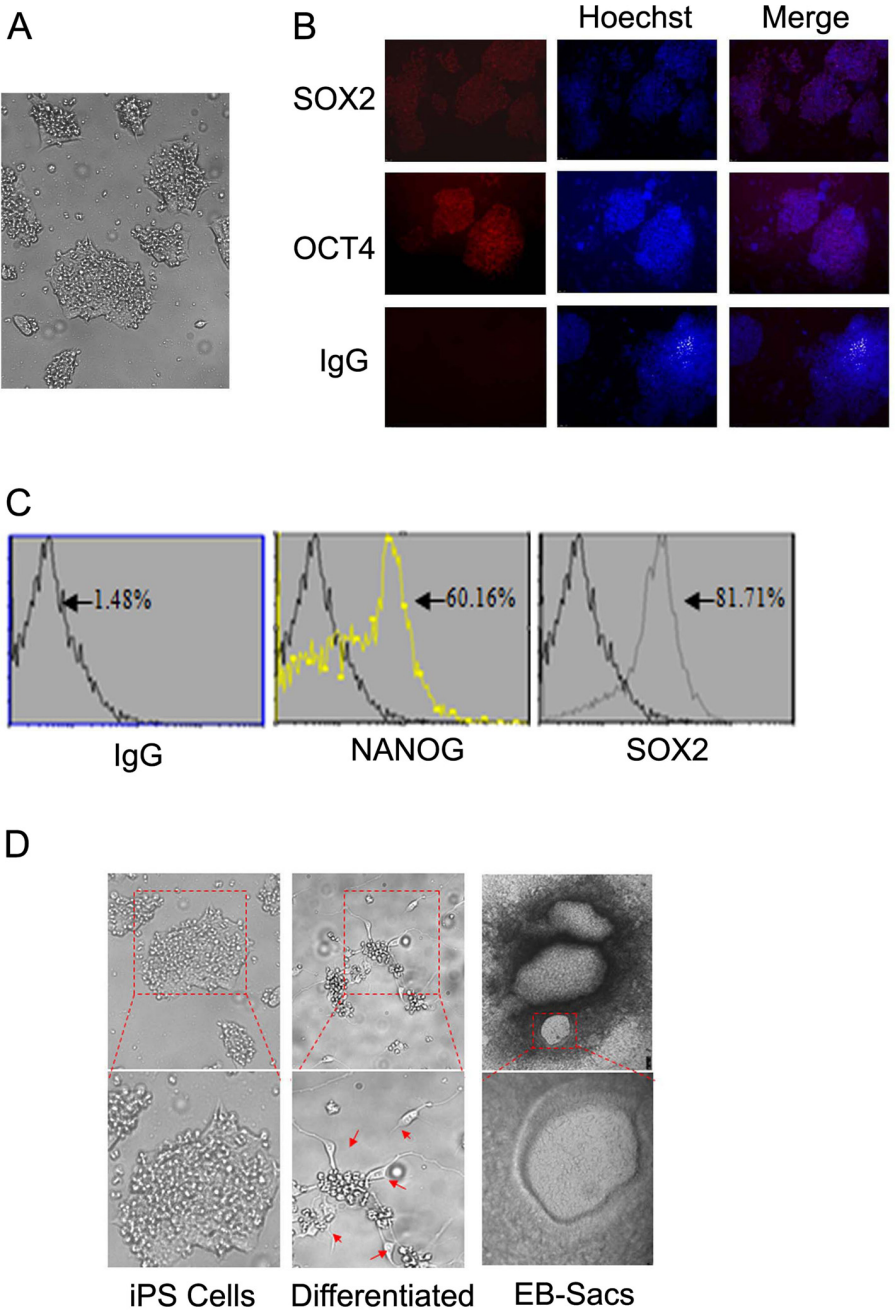
**Characterization of iPS cells. (A)** General morphology of iPS cells under study. **(B)** Human iPS cells were cultured on chamber slides, fixed and stained with either a control IgG or antibodies to SOX2 and OCT4. DNA was stained with Hoechst. Representative images are shown. **(C)** Human iPS cells were fixed and stained with either IgG or antibodies to NANOG and SOX2. Cells were then processed for analysis by flow cytometry. Experiments were repeated for at least three times. **(D)** Human iPS cells were cultured in a differentiation medium as described in Experimental procedures. Representative images of embryonic stem cell sacs (ES-sacs) and neural differentiation are shown.

Human iPS cells were obtained from amniotic fluid-derived cells after ectopic expression of four stem cell transcription factors (OCT4, SOX2, KLF4 and c-MYC) via the retroviral expression system. Microscopic examinations revealed that these cells exhibited a stem cell-like morphology and expressed abundant OCT4 and SOX2 (Figure 1A and B). Further characterization via flow cytometry indicated that these cells were also highly positive for NANOG and SOX2. We further examined these for their capacity for differentiation. Through incubation in a medium optimal for differentiation, we observed that these cells were capable of differentiating into cells with morphologic characteristics of neural cells or embryonic stem cell sacs (ES sacs) (Figure 1D). Combined, these results indicate that iPS cells under study bear essential characteristics of stem cells.

### Dose-dependent responses of human iPS and Tera-1 cells to Cr(VI)

**Figure 2:**
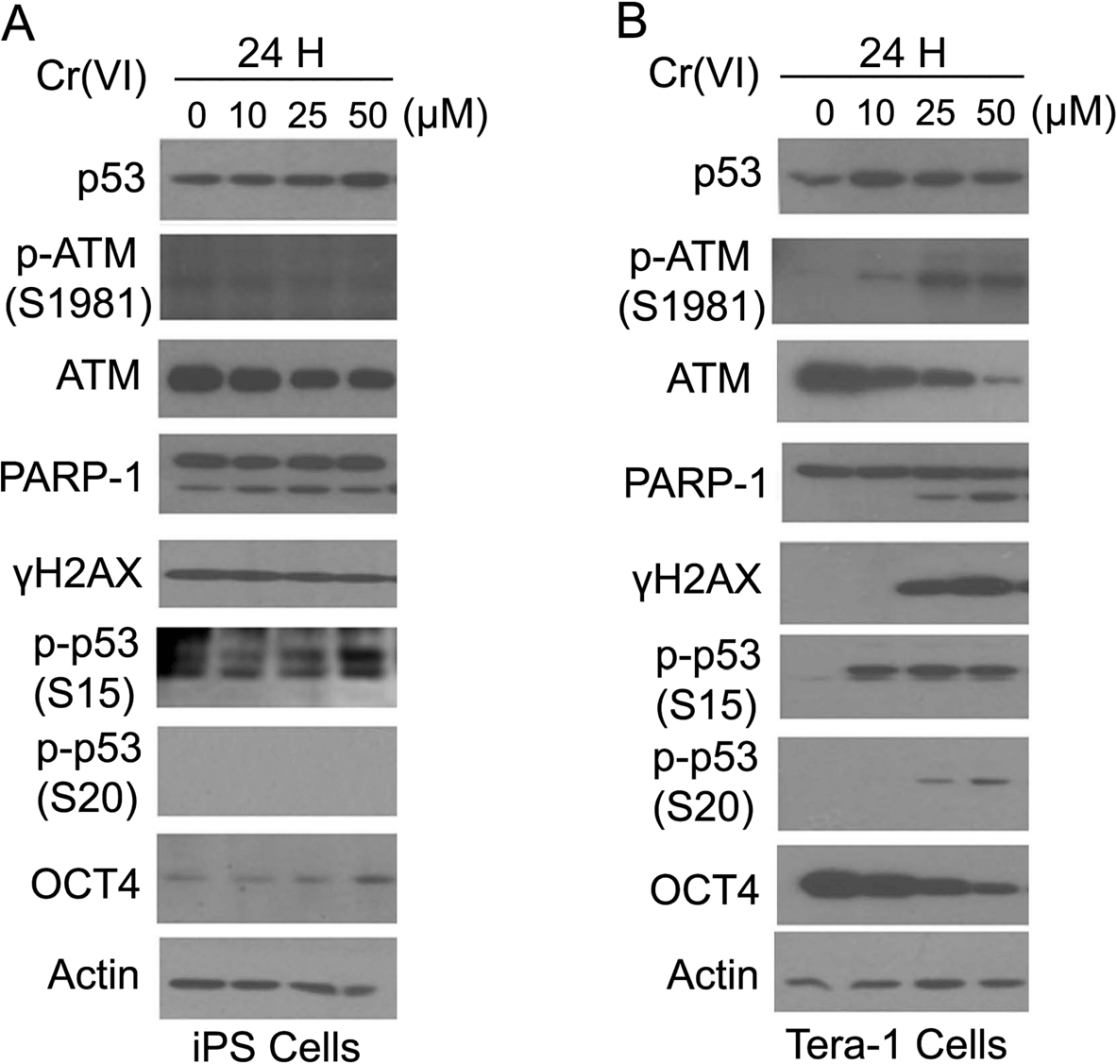
**Dose-dependent responses of human iPS and Tera-1 cells to Cr(VI). (A) **Human iPS cells were cultured with feeder cells as described in Experimental procedures and treated with Cr(VI) at various concentrations for 24 h. Whole cell lysates were then collected for Western blot analyses with various antibodies as indicated. **(B)** Tera-1 cells were cultured as indicated in Experimental procedures and treated with Cr(VI) for 24 h. Equal amounts of cell lysates were blotted with antibodies as indicated.

To study whether iPS cells were capable of responding to Cr(VI), we treated iPS cells, as well as Tera-1 cells as control, with the compound at various concentrations for 24 h. Western blot analysis revealed that p53 protein level displayed a slight increase after treatment with Cr(VI) in iPS cells (Figure 2A). We did not detect any significant induction of p-ATM^S1981^ (p-ATM thereafter) signals in human iPS cells (Figure 2A). In Tera-1 cells, p-ATM was induced by 10 μM Cr(VI), peaking at 25 μM (Figure 2B). In iPS cells, a relatively high basal level of γH2AX was present in untreated cells, which was not further increased after Cr(IV) treatment. In contrast, no basal level of γH2AX was detectable in Tera-1 cells and it was strongly induced by Cr(VI) at concentrations higher than 25 μM, which coincided with ATM phosphorylation/activation (Figure 2B). Phosphorylation of p53 on both S15 and S20 also exhibited a differential response to Cr(VI) treatment between iPS and Tera-1 cells. In iPS cells, p53 phosphorylation on S20 was not easily detectable whereas its phosphorylation on S15 was somewhat inducible. On the other hand, both S20 and S15 phosporylation in Tera-1 cells was strongly induced and S20 phosphorylation exhibited a dose-dependent increase after Cr(VI) treatment (Figure 2). The pattern of phosphorylation closely followed that of p-ATM signals. Combined, these data suggests that iPS cells are more resistant to Cr(VI)-induced DNA damage or that the DNA damage response pathway is suppressed in iPS cells. Intriguingly, expression of OCT4 in Tera-1 cells, but not in iPS cells, was suppressed by Cr(VI) (Figure 2A and B).

### Time-dependent response of human iPS, Tera-1, and BEAS-2B cells to Cr(VI)

We next determined the kinetics of phosphorylation/activation of various components in the DNA damage-response pathway. We treated iPS and Tera-1 cells with 10 μM Cr(VI) as this concentration elicited a minimal response of apoptosis while it activated ATM and p53 phosphorylation in Tera-1 cells (Figure 2B). We also treated BEAS-2B cells with Cr(VI) as an additional somatic cell control. In these experiments, iPS cells were cultured under feeder-free cultural conditions as opposed to the feeder-dependent condition in the previous studies so as to eliminate any potential interference of signals from the feeder cells.

**Figure 3:**
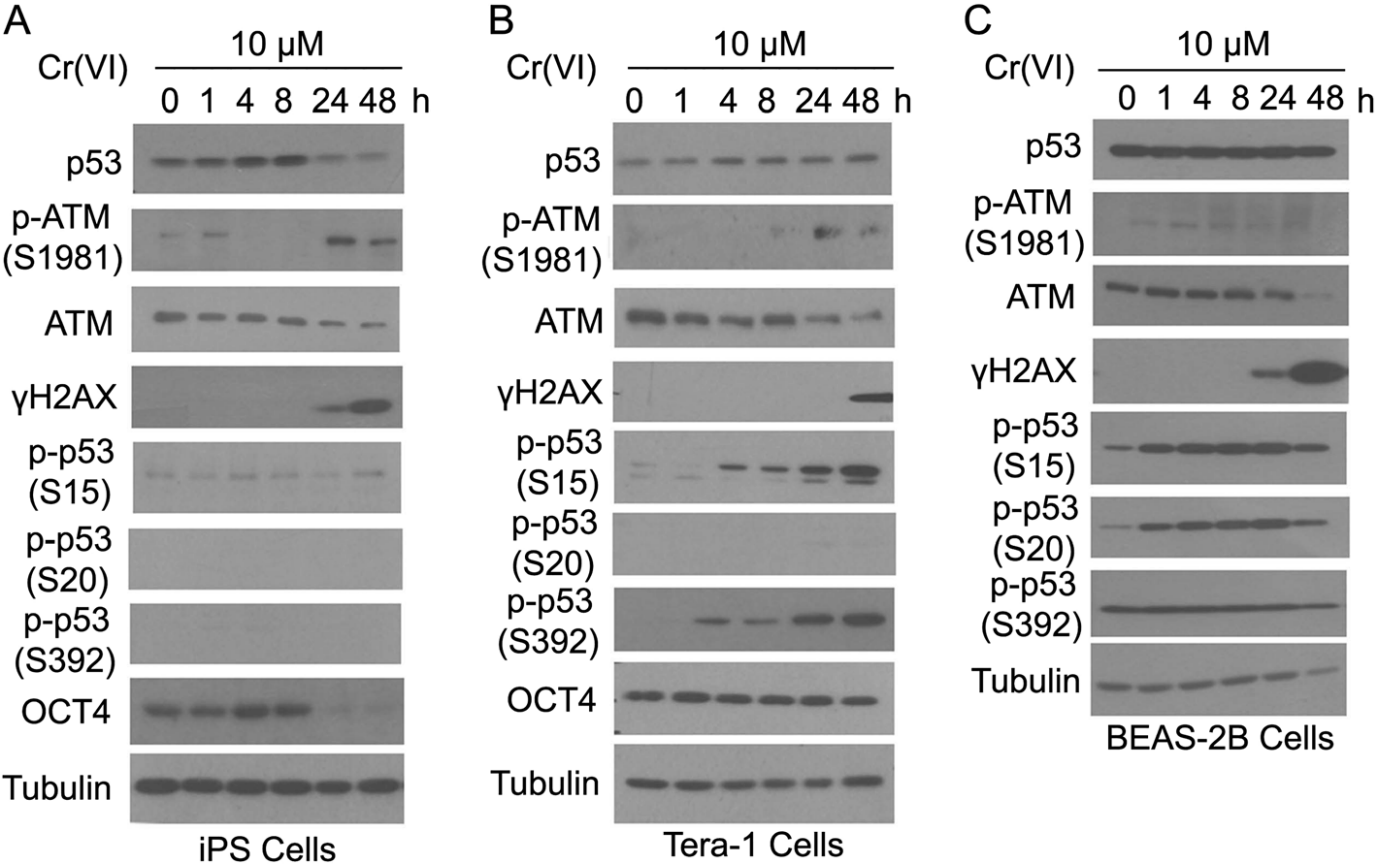
**Time-dependent responses of human iPS cells, Tera-1, and BEAS2B cells to Cr(VI). (A)** Human iPS cells were cultured under the feeder-free condition as described in Experimental procedures and treated with 10 μM Cr(VI) for various times as indicated. The whole cell lysates were collected and equal amounts of cell lysates were blotted with various antibodies as indicated. **(B)** Tera-1 cells were treated and analyzed as shown in **A**. **(C)** BEAS2B cells were treated and analyzed as shown in **A**.

There was a clear time-dependent increase in the p53 protein level in iPS cells and Tera-1 cells after Cr(VI) treatment (Figure 3A and B). A longer Cr(VI) treatment caused a decline of p53 in iPS cells. Interestingly, the p53 level in BEAS-2B was not significantly modulated during the treatment period (Figure 3C). The p-ATM level in iPS cells exhibited a biphasic pattern of induction after Cr(VI) treatment. It was slightly induced at about 1 h after treatment and then declined below the basal level until it increased again after cells were treated for more than 24 h (Figure 3A). The γH2AX signal was closely associated with the second phase of ATM phosphorylation in iPS cells. The p-ATM level in Tera-1 cells was only significantly elevated 24 h post Cr(VI) treatment, which was also correlated with the γH2AX signal (Figure 3B). Despite the non-detectable level of p-ATM in BEAS-2B cells, the γH2AX signal was increased in a manner similar to that in either iPS or Tera-1 cells.

Phosphorylation of p53 in three cells types was quite different. While the level of p-p53^S15^ in iPS cells did not show significant changes over time after Cr(VI) treatment, it was strongly induced in Tera-1 and BEAS-2B cells and its induction was time-dependent (Figure 3A-C). However, the p-p53^S15^ signal increased beyond 48 h in Tera-1 cells whereas it peaked at about 4 h post Cr(VI) treatment. In both iPS and Tera-1 cells, the level of p-p53^S20^ was undetectable before and after Cr(VI) treatments (Figure 3A and B). In contrast, p-p53^S20^ signal exhibited a time-dependent increase post treatment in BEAS-2B cells (Figure 3C). The phosphorylation level of p-p53^S392^, another important phosphorylation site in response to genotoxic stress [13], was distinctive among these three cell types. There were no detectable basal levels of p-p53^S392^ in iPS cells and Tera-1 cells (Figure 3A and B). However, it was inducible in Tera-1 cells after Cr(VI) treatment. There was a high basal level of p-p53^S392^ in BEAS-2B cells, which could not be further induced by exposure to Cr(VI).

### Time-dependent response of human iPS cells, Tera-1, and BEAS-2B cells to H_2_O_2_

**Figure 4:**
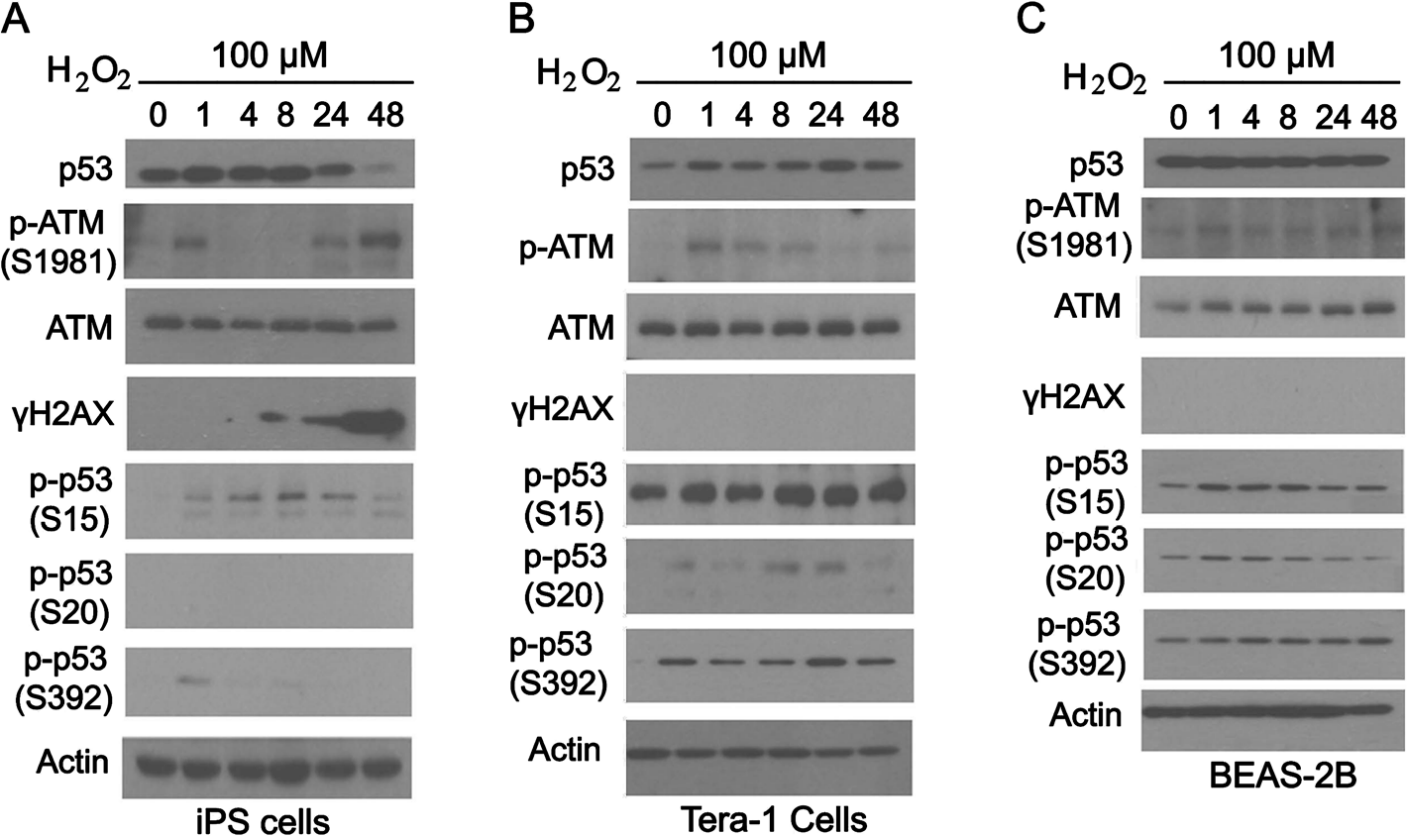
**Time-dependent responses of human iPS, Tera-1, and BEAS2B cells to H**_**2**_**O**_**2**_**. (A)** Human iPS cells were cultured under the feeder-free condition as described in Experimental procedures and treated with 100 μM H_2_O_2_ for the indicated time periods (h). The whole cell lysates were collected and equal amounts of lysates were blotted with antibodies to various cellular components as indicated. **(B)** Tera-1 cells were treated and analyzed as described in **A**. **(C)** BEAS-2B cells were treated and analyzed as described in **A**.

One of the main mechanisms for Cr(VI)-induced DNA damage responses is the generation of reactive oxygen species (ROS) [6-9]. We next examined the effect of H_2_O_2_ on the activation of key components of DNA damage responses in iPS, Tera-1 and BEAS-2B cells. We observed that different cell types responded to H_2_O_2_ in a manner similar to that of Cr(VI) with regard to p53 induction and activation (Figure 4). The total p53 level was slightly induced in iPS cells and was below the pretreatment level after 24 h treatment (Figure 4A). Whereas p53 was gradually induced in Tera-1 cells it was not significantly changed in BEAS-2B cells (Figure 4B and C). The p-ATM level in iPS cells treated with H_2_O_2_ displayed a biophasic pattern similar to that of Cr(VI) treatment (Figure 4A). In Tera-1 cells, however, p-ATM was induced most strongly at about 1 h of treatment (Figure 4B). This pattern was different from that of Cr(VI) treated Tera-1 cells, which only showed significant elevation of p-ATM at 24 h and 48 h time points (Figure 3B). In BEAS-2B cells, the p-ATM level was only slightly elevated over time after H_2_O_2_ treatment (Figure 4C), which is similar to the response to Cr(VI) (Figure 3C). The level of γH2AX in three cell types responded very differently to H_2_O_2_ compared with that in cells treated with Cr(VI). In iPS cells, it was strongly induced and the induction was time-dependent (Figure 4A). Surprisingly, we did not observe any induction of γH2AX in Tera-1 and BEASE-2B cells after H_2_O_2_ treatment (Figure 4B and C). This is in sharp contrast with the responses of these cells to Cr(VI), which was strongly induced at 48 h after Cr(VI) treatments (Figure 4B and C). In iPS cells, the level of p-p53^S15^ was significantly increased in a time-dependent fashion, peaking at 8 h of treatment (Figure 4A). Interestingly, H_2_O_2_ did not significantly induce p-p53^S15^ in either Tera-1 or BEAS-2B cells. Therefore, H_2_O_2_ behaves differently in induction of p-p53^S15^ compared with that of Cr(VI). Similar to Cr(VI), H_2_O_2_ did not induced any p-p53^S20^ (compare Figures 3A and 4A). However, the level of p-p53^S20^ in Tera-1 cells was elevated in response to H_2_O_2_ treatments (Figure 4B). This was in contrast to the response to Cr(VI), which showed no increase (Figure 3B). In Tera-1 cells, p-p53^S392^ levels were rapidly induced by H_2_O_2_ and remained elevated throughout the time course, which was different from the time-dependent increase shown in Cr(VI) treatments (Figure 3B with Figure 4B). In BEAS-2B cells, however, the kinetics of p-p53^S392^ level after H_2_O_2_ treatment was similar to that of Cr(VI) (Figure 3C with Figure 4C).

### Time-dependent response of human iPS cells, Tera-1, and BEAS-2B cells to Dox

**Figure 5:**
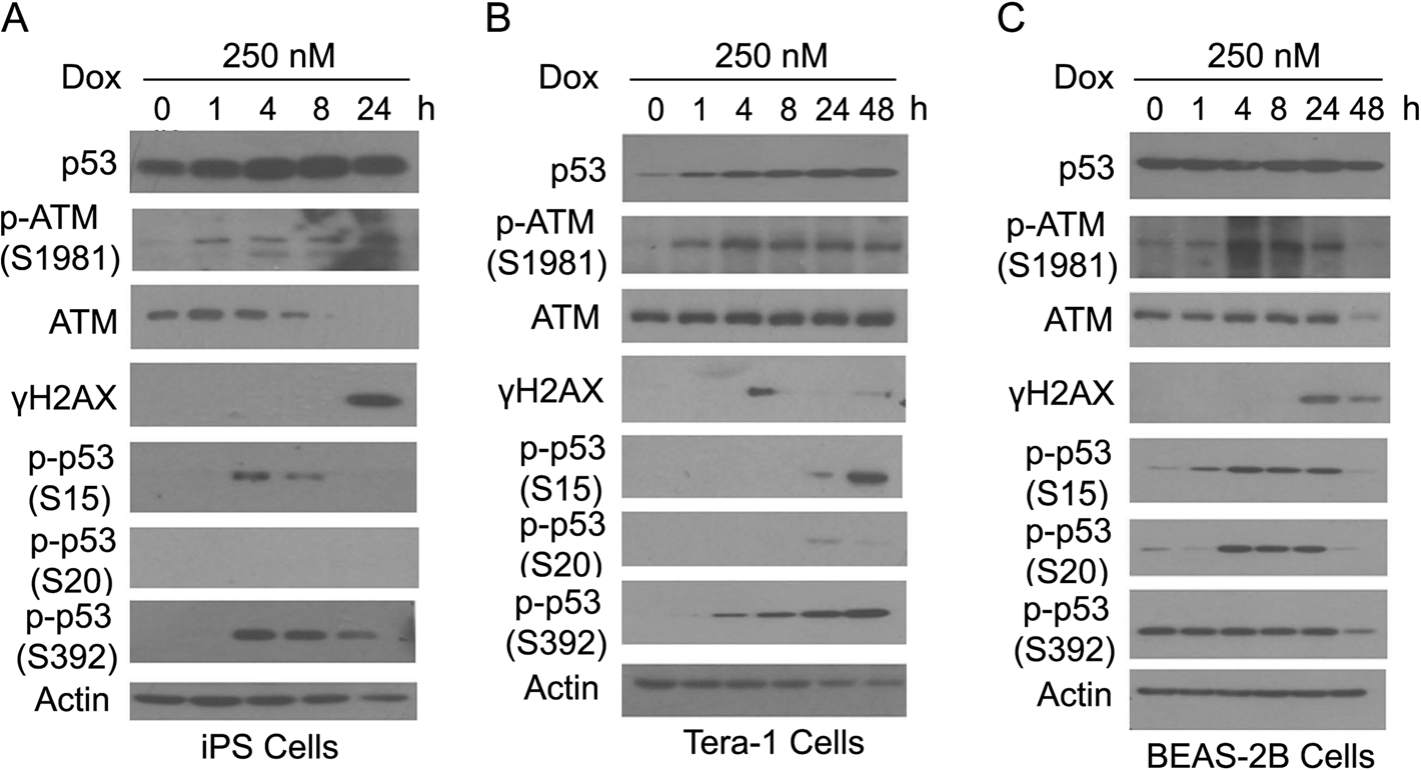
**Time-dependent responses of iPS, Tera-1, and BEAS2B cells to Doxorubicin. (A)** Human iPS cells were cultured under the feeder-free condition as described in Experimental procedures and treated with 250 nM Dox for the indicated times. Whole cell lysates were collected and equal amounts of lysates were blotted with various antibodies as indicated. **(B)** Tera-1 cells were treated and analyzed as described in **A**. **(C)** BEAS-2B cells were treated and analyzed as described in **A**.

Given that doxorubicin (Dox) is capable of inducing double strand breaks [19,20], we further analyzed DNA damage responses in three different cell types after treatment with this chemical compound. Again, we observed that general patterns of total p53 levels in response to Dox were similar to those induced by either Cr(VI) or H_2_O_2_. A steady increase of p53 was observed in both iPS and Tera-1 cells whereas it was largely unchanged in BEAS-2B cells (Figure 5). In contrast to Cr(VI) and H_2_O_2_, Dox induced p-ATM in a time-dependent fashion in iPS cells (Figure 5A). The level of p-ATM in Tera-1 and BEAS-2B cells after Dox treatment was similar to that of H_2_O_2_ (Figure 5B). It rapidly increased, peaking at about 4 h post treatment followed by a gradual decline to the pretreatment level (Figure 5B and C). Gamma H2AX signals in iPS and BEAS-2B cells were strongly induced after extended treatment with Dox (Figure 5A and C). Intriguingly, γH2AX signals in Tera-1 cells were transiently induced at about 4 h post treatment (Figure 5B). Different from Cr(VI), Dox was capable of inducing p-p53^S15^ in iPS cells (Figure 5A). On the other hand, Dox induced p-p53^S15^ only after 24 h of treatment (Figure 5B), which is very different from that in cells treated with Cr(VI) or H_2_O_2_. Phosphorylation of p53 on S20 was only strongly induced by Dox in BEAS-2B cells whereas phosphorylation on S392 was induced in both iPS and Tera-1 cells, but not in BEAS-2B cells. Table 1 summarizes the time-dependent responses of the three cell lines to three different types of genotoxic agents.

**Table 1:** **Time-dependent responses of human iPS, Tera-1, and BEAS-2B cells after treatment with Cr(VI), H**_**2**_**O**_**2, **_**or Dox**

**Cell type**	**Treatment**	**Total p53**	**p-ATM**	**γH2AX**	**p-p53**^**S15**^	**p-p53**^**S20**^	**p-p53**^**S392**^
iPS cells	Cr(VI)	Up early down late	Biphasic induction	Strongly up late	Unchanged	Undetectable	Undetectable
	10 μM						
	H_2_O_2_	Slightly up early and down late	Biphasic induction	Strongly up late	Strongly up early and down late	Undetectable	Slightly up early and down late
	100 μM						
	Dox	Slightly up over time	Up over time	Up late	Up early and down late	Undetectable	Strongly up early and down late
	250 nM						
Tera-1	Cr(VI)	Slightly up over time	Up late	Strongly up late	Strongly up overtime	Undetectable	Strongly up over time
	10 μM						
	H_2_O_2_	Slightly up over time	Up early and down late	Undetectable	Unchanged	Up over time and down late	Up early and stays up
	100 μM						
	Dox	Significantly up over time	Significantly up early and down late	Strongly up at 8 h and down late	Strongly up late	Slightly up late	Strongly up over time
	250 nM						
BEASE-2B	Cr(VI)	Unchanged	Slightly up over time	Strongly up late	Up over time	Up over time	Unchanged
	10 μM						
	H_2_O_2_	Unchanged	Largely unchanged	Undetectable	Largely unchanged	Largely unchanged	Largely unchanged
	100 μM						
	Dox	Unchanged	Up early and down late	Strongly up late	Strongly up early and down late	Strongly up early and down late	Unchanged up to 24 h and down at 48 h
	250 nM						

### Roles of p38 and CK2 on p53^S392^ phosphorylation

**Figure 6:**
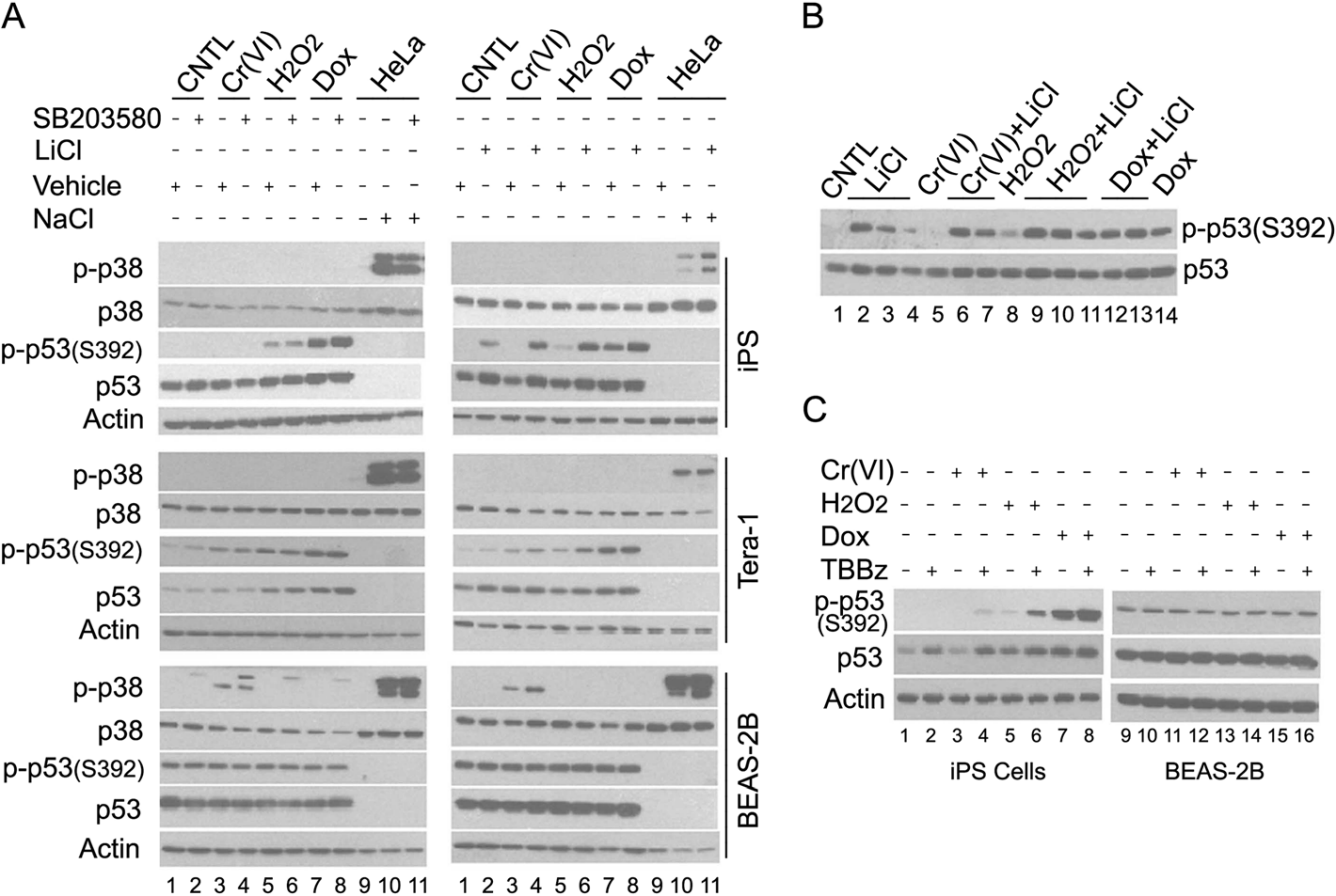
**Roles of p38 and CK2 on p53**^**S392 **^**phosphorylation. (A)** Human iPS, Tera-1, or BEAS-2B cells were treated with 10 μM Cr(VI), 100 μM H_2_O_2,_ or 250 nM Dox for 4 h with or without pretreatment with 10 μM SB203580 or 10 μM LiCl for one h. HeLa cells treated with 500 mM NaCl was used as a positive control for p38 activation. Whole cell lysates were collected and equal amounts of cell lysates were blotted with antibodies to various molecular components as indicated. **(B)**. Different amounts of human iPS cell lysates from experiments described in **(A)** were used for Western blot to match (or normalize) the total p53 level as indicated. The p-p53^S392^ level was detected using the antibody to p-p53^S392^. **(C)**. Human iPS or BEAS-2B cells were treated with 10 μM Cr(VI), 100 μM H_2_O_2,_ or 250 nM Dox for 4 h with or without pretreatment with 10 μM TBBz for one h. The whole cell lysates were collected and equal amounts of cell lysates were blotted with antibodies to various molecular components as indicated.

The striking difference in p53^S392^ phosphorylation between three cell types in response to different stimuli prompted us to further explore on the potential kinases that phosphorylate this residue in these cells. Previous studies have shown that the p38 MAP kinase and casein kinase 2 (CK2) are the main kinases that phosphorylate p53^S392^[21-23]. We examined whether these two kinases are responsible for the observed differential phosphorylation of this residue. As shown in Figure 6A (lanes 1–8), p38 was not activated by these genotoxic agents as indicated by the p-p38 level. The specific p38 inhibitor SB203580 had no effect on the phosphorylation of p53^S392^ induced by H_2_O_2_ or Dox in iPS or Tera-1 cells. In BEAS-2B cells, p38 was only activated by Cr(VI). However, the high basal level of p-p53^S392^ in BEAS-2B cells, which was not induced by the genotoxic agents, was not affected by p38 inhibition. HeLa cells treated with 500 mM NaCl were used here as a positive control for p38 activation (lanes 9–11). These data indicate that p38 is unlikely the kinase that phosphorylated p53^S392^ in these cells before and after exposure to these genotoxic agents.

To determine whether CK2 was responsible for p53^S392^ phosphorylation, we first treated cell with LiCl, which significantly inhibits CK2 [24]. As shown in Figure 6 (lanes 1–8, right panels), LiCl treatment led to increased levels of p53^S392^ phosphorylation with or without treatment with genotoxic agents in iPS cells. In contrast, LiCl had a limited effect on p53^S392^ phosphorylation in Tera-1 cells. The high basal level of p-p53^S392^ in BEAS-2B cells was not affected by LiCl treatment either. Since the elevated p-p53^S392^ level induced by LiCl in iPS cells appears to correlate with the increased level of total p53, we normalized the level of total p53 to see if the difference in the p-p53^S392^ level still exists. As shown in Figure 6B, the elevated p-p53^S392^ level remains after normalization of total p53 among treatments, indicating that LiCl indeed promotes the phosphorylation of p53^S392^ in iPS cells.

We next treated cells with 4,5,6,7-tetrabromobenzimidazole (abbreviated as TBBz), a specific inhibitor of CK2 [25], to determine whether phosphorylation of p53^S392^ is at least partly mediated by CK2 in iPS cells. As shown in Figure 6C, TBBz treatment significantly enhanced phosphorylation of p53^S392^ in iPS cells, which is similar to the effect observed after LiCl treatment in these cells. Combined, these results strongly suggest that CK2 inhibition promotes phosphorylation of p53^S392^ in iPS cells before and after exposure to genotoxic agents.

## Discussion

The current study surveys the activation of several key elements of DNA damage response pathways in human iPS, Tera-1 cells, and BEAS-2B cells after treatment with Cr(VI), a well documented genotoxic agent. We also compared the responses of those cells to other DNA damaging agents including H_2_O_2_ and Dox. Our results indicate that iPS cells possess a rather distinct pattern of responses compared with established cell lines. While Cr(VI) induces ATM phosphorylation in a unique biphasic fashion and increases the level of γH2AX after prolonged treatments in iPS cells it is largely unable to elicit phosphorylation of p53 on S15, S20, and S392 in these cells (Figure 3A and Table 1). In contrast, the level of p-p53^S15^ is easily inducible by H_2_O_2_ and Dox and p53^S392^ is strongly induced by Dox in iPS cells (Figures 4A, and 5A). The p-p53^S20^ level in iPS cells seems to be rather resistant to genotoxic insults as it fails to respond to any of genotoxic agents under study. Compared with iPS cells, p53 phosphorylation patterns on these residues in Tera-1 and BEAS-2B cells are rather different after Cr(VI) treatment. A gross comparison of the responses of three cell types to these different categories of genotoxic agents reveals some interesting findings: (i) While p-p53^S20^ is easily inducible by all three agents in BEAS-2B cells it is much less responsive, if at all, in iPS cells and Tera-1 cells; (ii) Although the level of p-p53^S392^ in BEAS-2B cells is constitutively elevated it is irresponsive to these genotoxic compounds. On the other hand, its phosphorylation is elicited in iPS cells and strongly induced in Tera-1 cells by these compounds.

Phosphorylation of p53 on S15 is known to stabilize the transcription factor [15,16]. Extensive research in the past has shown that this residue is the target of multiple kinases, including ATM and ATR [15,16] whereas S20 is the target of MAPKAPK2, JNK, CHK2 and Plk3 [15,16]. Consistent with these studies, we also show that ATM is phosphorylation on S1981 after treatment with various genotoxic agents, correlating with p53 and γH2AX phosphorylation. Phosphorylation of p53 on S15 and S20 is believed to an integral part of the DNA damage response *in vivo*[15,16]. The relative resistance of S20 phosphorylation to DNA damaging agents under study in iPS and Tera-1 cells is intriguing as both types of cells possess stem cell-like characteristics (Figures 1 and 2) [1,26]. It is tempting to speculate that stem cells may be lack of components that lead to p53 phosphorylation on S20 or that highly activated p53 may not be ideal for stem cell self-renewal. Supporting this notion, p53 levels adversely affect reprogramming iPS cells from somatic cells [27].

It has been shown that phosphorylation of p53 on S392 is mediated by p38, CK2, PKR, and CDK9 [22,28-30] although a recent study suggests that this residue is not a target of p38 or CK2 but rather of a yet unknown kinase [23]. Moreover, phosphorylation of this residue appears to respond to diverse stimuli that induce p53 [23], suggesting that S392 phosphorylation may play a broad role in the regulation p53. We have observed that p-p53^S392^ signals display rather distinctive profiles in three cell types tested. Notably, p-p53^S392^ levels are very low in iPS and Tera-1 cells. While p-p53^S392^ in Tera-1 cells is highly induced by all three genotoxic agents it is only significantly induced by Dox treatment in iPS cells. On the other hand, p-p53^S392^ levels are high and hardly respond to any type of stimuli under study. These dramatic differences of p-p53^S392^ between these cells reflect a complex regulation of this residue in a cell type-dependent and/or stimulus-dependent fashion. Our study with specific chemical inhibitors revealed that p38 is not involved in p53^S392^ phosphorylation in all three cell types with or without genotoxic insults (Figure 6A). CK2 also seems to be not involved in p53^S392^ phosphorylation in Tera-1 and BEAS-2B cells either (Figure 6). These results are largely in agreement with the previous study [23]. Surprisingly, CK2 appears to inhibit p53^S392^ phosphorylation in iPS cells as inhibition of the enzyme strongly enhances p-p53^S392^ signals (Figure 6). This observation is very interesting as previous studies show that CK2 can promote, rather than inhibit, p53 phosphorylation on S392 [31]. Therefore, further investigation on how CK2 inhibits p53^S392^ may help to unravel a novel mechanism of p53 regulation in stem cells after exposure to genotoxic stress. Of note, in all the three cell types studied, p53^S392^ phosphorylation is very closely and positively correlated with the total p53 level (Figure 6A). Given that small molecules have been developed for enhancing p53 activity for the treatment of various cancers [32], our further study of differential responses in stem cells and somatic cells can lead to the discovery of new targets for p53 activation.

The main mechanism of Cr(VI)-mediated DNA damage responses is believed to be through the generation of oxidative stress [6-9]. H_2_O_2_ can directly produce oxidative stresses. Dox inhibits the action of topoisomerase II and also generates oxidative stress, thereby triggering DNA damage response [19,20]. Despite the overlapping mechanisms of action of these genotoxic agents, differential responses as revealed by the current study suggest that each agent may activate different signaling components of the DNA damage response network. Thus, the effect of Cr(VI) on cellular responses may not be just limited to the generation of oxidative stresses. Whether the unique chromatin structures in iPS cells (stem cells) may contribute to the differential responses remains to be determined.

## Competing interest

The authors declare that they have no competing interests.

## Authors’ contributions

YL: She carried out experiments and analyzed data. DX: He designed experiments, analyzed data and was involved in manuscript writing. JZ: She was involved in designing and performing experiments. YM: He helped experimental designs and provided some key reagents. YJ: He helped in data interpretations and provided key reagents. WZ: He helped in experimental designs and data interpretation. WD: He designed experiments, analyzed data, and write the manuscript. All authors read and approved the final manuscript.
